# Bone-seeking TRAP conjugates: surprising observations and their implications on the development of gallium-68-labeled bisphosphonates

**DOI:** 10.1186/2191-219X-2-13

**Published:** 2012-03-30

**Authors:** Johannes Notni, Jan Plutnar, Hans-Jürgen Wester

**Affiliations:** 1 Pharmaceutical Radiochemistry, Technische Universität München, Walther-Meissner-Str. 3, Garching 85748, Germany; 2 Department of Inorganic Chemistry, Faculty of Science, Charles University in Prague, Hlavova 2030, Prague 2 12843, Czech Republic

**Keywords:** Gallium-68, Bisphosphonates, Positron emission tomography, Bone seekers, MicroPET, Bone imaging

## Abstract

**Background:**

Bisphosphonates possess strong affinity to bone. ^99m^Tc bisphosphonate complexes are widely used for bone scintigraphy. For positron emission tomography (PET) bone imaging, Ga-68-based PET tracers based on bisphosphonates are highly desirable.

**Findings:**

Two trimeric bisphosphonate conjugates of the triazacyclononane-phosphinate (TRAP) chelator were synthesized, labeled with Ga-68, and used for microPET imaging of bone in male Lewis rats. Both Ga-68 tracers show bone uptake and, thus, are suitable for PET bone imaging. Surprisingly, Ga-71 nuclear magnetic resonance data prove that Ga(III) is not located in the chelating cavity of TRAP and must therefore be bound by the conjugated bisphosphonate units.

**Conclusion:**

The intrinsic Ga-68 chelating properties of TRAP are not needed for Ga-68 PET bone imaging with TRAP-bisphosphonate conjugates. Here, TRAP serves only as a trimeric scaffold. For preparation of Ga-68-based bone seekers for PET, it appears sufficient to equip branched scaffolds with multiple bisphosphonate units, which serve both Ga-68-binding and bone-targeting purposes.

## Background

**Figure 1:**
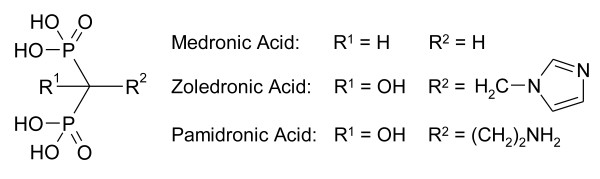
**Examples for common bisphosphonate drugs**.

**Figure 2:**
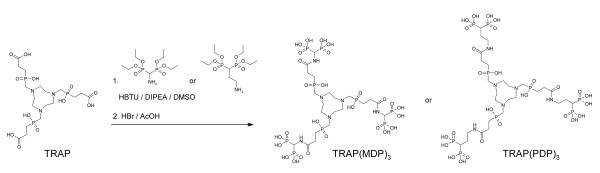
**Synthesis of trimeric TRAP-bisphosphonate conjugates TRAP(MDP)_3 _and TRAP(PDP)_3_**.

Geminal bisphosphonates possess strong affinity to bone [1,2]. In living organisms, administration of bisphosphonates leads to inhibition of osteoclasts (bone resorbing cells), which results in a lower rate of bone resorption [3,4]. Therapy with bisphosphonate drugs is thus performed to prevent decrease of bone density caused by osteogenesis imperfecta (brittle bone disease) [5] or osteoporosis [3,6]. In addition, bisphosphonate complexes of ^99m^Tc (e.g., of medronic acid, '^99m^Tc-MDP'; see Figure 1) are the mainstay of bone imaging by scintigraphy and SPECT. However, as positron emission tomography (PET) offers higher resolution and sensitivity, PET bone-imaging agents are of high interest. Direct utilization of the β^+^-emitting radionuclide ^18 ^F (*t*_1/2 _= 110 min, *E*_max,β+ _= 634 keV) is the most simple and straightforward approach because [^18 ^F]fluoride ^18 ^F^- ^inherently possesses a high affinity to bone. However, ^18 ^F is cyclotron-produced, and therefore, a full geographical coverage, comparable to the supply of generator-produced ^99m^Tc, cannot be guaranteed. Thus, bisphosphonate mono-conjugates of the currently most popular radiometal chelators 1,4,7,10-tetraazacyclododecane-tetraacetic acid [7-9] and 1,4,7-triazacyclononane-triacetic acid [10] have been prepared to utilize generator-produced ^68 ^Ga (*t*_1/2 _= 68 min, *E*_max,β+ _= 1.9 MeV) for PET bone imaging. Advancing this approach, this pilot study describes preclinical PET imaging results for trimeric bisphosphonate conjugates of the recently introduced chelator triazacyclononane-phosphinate (TRAP) (see Figure 2) [11-13].

## Methods

General procedures and instrumentation (nuclear magnetic resonance (NMR), electrospray mass spectroscopy (ESI-MS), ultrafiltration/diafiltration, PET) have been described before [13]. [^18 ^F]fluoride formulation for injection was prepared by adding 100 MBq of ^18 ^F (obtained from routine cyclotron production at Klinikum rechts der Isar, Technische Universität München, München, Germany) to phosphate buffered saline (PBS) (1 mL).

Synthesis of bisphosphonate conjugates (Figure 2): TRAP·2H_2_O (0.3 mmol, 185 mg), diisopropylethylamide (3 mmol, 388 mg, 510 μL), and the amino-bisphosphonate (1.5 mmol); for TRAP(MDP)_3_, tetraethyl(aminomethylene)bisphosphonate 455 mg; and for TRAP(PDP)_3_, tetraethyl(1-aminopropylene)bisphosphonate 500 mg, were dissolved in DMSO (2 mL). Then, HATU (2.4 mmol, 921 mg) was added with stirring. After 25 min, the reaction mixture was diluted with water (50 mL) and subjected to diafiltration (membrane with 500 Da MWCO). After 250 mL of water had passed, the cell content was concentrated *in vacuo *and subjected to preparative HPLC (column: YMC C18 ec 250 × 30 mm; detection wavelength, 220 nm; eluent A, MeCN with 0.1% TFA; eluent B, water with 0.1% TFA; gradient 25% to 50% B in 20 min, *t*_R_(dodecaethyl-TRAP(MDP)_3_) = 12 min, *t*_R_(dodecaethyl-TRAP(PDP)_3_) = 16 min). After evaporation of the solvents, the remaining viscous oil was dissolved in HBr/glacial acetic acid (33%) and stirred for 3 days. Addition of methanol to the reaction mixture yielded the products as colorless, crystalline solids. Data for TRAP(MDP)_3_: yield 201 mg (61%); MW (calculated for C_21_H_51_N_6_O_27_P_9_) 1,098.43; ESI-MS negative *m/z *= 1,097 (M-H^+^) and 548 (M-2 H^+^); ^1 ^H NMR (600 MHz, D_2_O) *δ *= 2.13 (m, 6 H), 2.67 (m, 6 H), 3.48 (d, ^3^*J*_HH _= 5.4 Hz, 6 H), 3.56 (s, broad, 12 H), and 4.71 (t, *J*_PH _= 21.3 Hz, 3 H) ppm; ^13 ^C NMR (151 MHz, D_2_O) *δ *= 26.11 (d, ^1^*J*_PC _= 93.18 Hz), 28.65, 52.13, 54.74 (d, ^1^*J*_PC _= 89.07 Hz), 47.45 (t, ^1^*J*_PC _= 139.28 Hz), and 174.54 (dt, ^2^*J*_PC _= 12.28 and ^3^*J*_PC _= 4.34 Hz) ppm; and ^31^P NMR (121 MHz, D_2_O) *δ *= 14.10 (d, ^2^*J*_PP _= 15.7 Hz) and 39.90 ppm. Data for TRAP(PDP)_3_: yield 195 mg (55%); MW (calculated for C_27_H_63_N_6_O_27_P_9_) 1,182.59; ESI-MS negative *m/z *= 1,181 (M-H^+^), 590 (M-2 H^+^), and 393 (M-3 H^+^); ^1 ^H NMR (600 MHz, D_2_O) *δ *= 2.07 (m, 6 H), 2.10 (m, 6 H), 2.36 (tt, *J*_PH _= 23.52 Hz, ^3^*J*_HH _= 5.97 Hz, 3 H), 2.53 (m, 6 H), 3.44 (t, ^3^*J*_HH _= 6.3 Hz, 6 H), 3.45 (t, broad, ^3^*J*_HH _= 6.6 Hz), and 3.52 (s, broad, 12 H) ppm; ^13 ^C NMR (151 MHz, D_2_O) *δ *= 25.36 (t, ^2^*J*_PC _= 4.2 Hz), 26.29 (d, ^1^*J*_PC _= 93.5 Hz), 28.63 (d, ^2^*J*_PC _= 3.9 Hz), 35.77 (t, ^1^*J*_PC _= 128.5 Hz), 39.48 (d, ^3^*J*_PC _= 7.4 Hz), 52.14, 54.82 (d, ^1^*J*_PC _= 88.6 Hz), and 175.41 (d, ^3^*J*_PC _= 13.1 Hz) ppm; and ^31^P NMR (121 MHz, D_2_O) *δ *= 21.53 (d, ^2^*J*_PP _= 15.5 Hz) and 39.69 ppm.

^68 ^Ga for labeling was obtained from a SnO_2_-based ^68^Ge/^68 ^Ga generator (iThemba LABS, Somerset West, South Africa), eluted with 1.0 M HCl. A 1.25 mL fraction of the eluate containing *ca*. 80% of the total activity (*ca*. 1.3 GBq) was adjusted to pH 3.3 by adding a solution of 600 mg 2-[4-(2-hydroxyethyl)-1-piperazinyl]-ethanesulfonic acid (HEPES) in 0.5 mL water. To a 90 μL aliquot of this mixture, 10 μL of 10^-4 ^M stock solution of the ligand was added. After heating for 5 min to 95°C, the solution was passed over a cation exchanger SPE cartridge (Chromafix HR-XC M, Macherey-Nagel, Düren, Germany) and purged with water (1 mL). This procedure removed non-complexed ^68 ^Ga as well as HEPES, which was confirmed by processing of blank samples. Radiochemical yields, determined by measuring the activity on the cartridge and in the eluate, were > 85%. Formulation was done by adjusting the pH of the eluate to 7.4 by adding approximately 50 μL of a solution of NaOH (1 g) and NaH_2_PO_4 _(483 mg) in water (20 mL) while monitoring the pH with a pH meter. 'Free' ^68 ^Ga formulation was prepared by addition of the generator eluate (40 μL, *ca*. 50 MBq) to PBS (1 mL), resulting in pH 7.2.

All animal experiments were carried out in accordance with the current animal welfare regulations in Germany. Five male Lewis rats (age 7 weeks, *ca*. 200 g) were kept under standard laboratory conditions (12 h light/12 h dark) and given standard diet and water *ad libitum*. For PET, *ca*. 35 MBq of tracer was injected into the tail vein under isoflurane anesthesia. Two subsequent scans of 15 min were recorded 60 min post injection, using two different axial bed positions in order to image the entire animals. Images were reconstructed using a OSEM3D algorithm without scatter and attenuation correction. For each full-body maximum intensity projection (MIP), two part-body MIPs were stitched together manually using graphics software. PET images are from representative animals reflecting the group.

## Results and discussion

**Figure 3:**
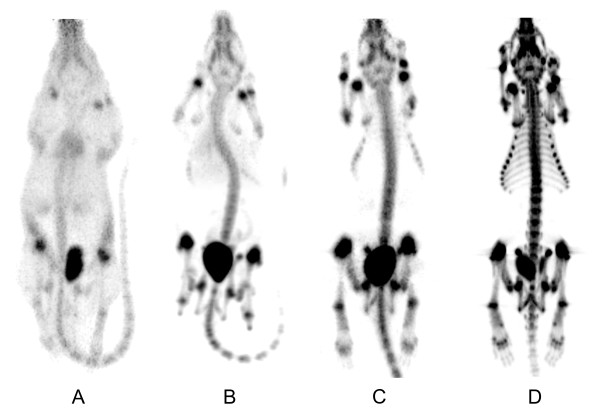
**PET scans (MIP, 60 min p.i.) of Lewis rats using different tracers**. **(A**) Free ^68 ^Ga(III), (**B**) ^68 ^Ga-TRAP(MDP)_3_, (**C**) ^68 ^Ga-TRAP(PDP)_3_, and (**D**) [^18 ^F]fluoride.

Figure 3 shows that free ^68 ^Ga(III) (we use this generalized term since ^68 ^Ga species in PBS solutions are not well defined) provides almost no contrast of the skeleton over other tissues, as intravenous injection of free ^68 ^Ga(III) predominantly results in transferrin-bound activity [14-17]. In contrast, both bisphosphonate tracers ^68 ^Ga-TRAP(MDP)_3 _and ^68 ^Ga-TRAP(PDP)_3 _bind to bone while showing low levels in blood and soft tissues. Apparently, PET image quality achieved therewith cannot compete with that of [^18 ^F]fluoride. ^18 ^F possesses a lower positron energy than ^68 ^Ga, resulting in lower tissue penetration (FW20H of 0.54 mm and 2.12 mm in soft tissue for ^18 ^F and ^68 ^Ga, respectively [18]), and therefore in a lower degree of image blurring. However, as the difference in resolution for a clinical 3-mm PET camera is small (3.05 mm for ^18 ^F and 3.57 mm for ^68 ^Ga [18]), a successful application of ^68 ^Ga bone-imaging agents in patients is not precluded.

Upon investigation of the mode of gallium binding, we found that an equimolar mixture of ^69,71 ^Ga^3+ ^and either ^68 ^Ga-TRAP(MDP)_3 _or ^68 ^Ga-TRAP(PDP)_3 _does not yield any signal in ^71 ^Ga NMR spectra, not even after heating to 95°C for hours. However, the octahedral N_3_O_3 _coordination usually found for 'in-cage' Ga(III) complexes of TRAP ligands generally yields sharp ^71 ^Ga NMR resonances at *δ *= 130 to 142 ppm [11,12]. Obviously, Ga(III) ion is not located in the TRAP cavity and, therefore, must be complexed in an 'out-of-cage' manner by the bisphosphonate groups. Although this result is quite unexpected, PET images nevertheless prove that the degree of kinetic stability of these complexes is sufficiently high to carry ^68 ^Ga to the bone and retain it there. However, Figure 3 also shows a slightly higher background uptake for ^68 ^Ga-TRAP(MDP)_3_, most likely caused by partial decomplexation *in vivo *due to lower complex stability. Clearance of both ^68 ^Ga tracers occurred faster than ^18 ^F^- ^and exclusively via the kidneys.

## Conclusion

^68 ^Ga-labeled trimeric bisphosphonate conjugates of TRAP were successfully applied for bone imaging in rats. Surprisingly, ^71 ^Ga NMR investigation revealed that Ga(III) ion is not located in the macrocyclic cavity of TRAP and, therefore, must be complexed by one or more side chain bisphosphonates. Although the primary chelation site of TRAP possesses excellent Ga(III) complexing properties [12], it apparently cannot compete with the bisphosphonates. In ^68 ^Ga-TRAP(MDP)_3 _and ^68 ^Ga-TRAP(PDP)_3_, TRAP thus merely serves as a scaffold, and its ability for ^68 ^Ga binding is not required. We therefore conclude that in designing bisphosphonate tracers for ^68 ^Ga-based PET bone imaging, the introduction of chelating moieties other than the bisphosphonates themselves might be unnecessary. Rather, it appears to be sufficient to equip suitable branched scaffolds with multiple bisphosphonate units which serve both ^68 ^Ga-binding and bone-targeting purposes.

## Abbreviations

ESI-MS: electrospray mass spectroscopy; HEPES: 2-[4-(2-hydroxyethyl)-1-piperazinyl]-ethanesulfonic acid; MIP: maximum intensity projection; NMR: nuclear magnetic resonance; PBS: phosphate buffered saline; PET: positron emission tomography; TRAP: triazacyclononane-phosphinate.

## Competing interests

The authors declare that they have no competing interests.

## Authors' contributions

JN developed the study concept; performed synthesis, radiolabeling, PET imaging, and PET data analysis; and wrote the manuscript. JP performed all NMR measurements and evaluated the data. HJW provided important advice in the conception of the study, gave advice in the interpretation of the data, and critically reviewed the manuscript. All authors read and approved the final manuscript.
